# Cognitive function is associated with home discharge in subacute stroke patients: a retrospective cohort study

**DOI:** 10.1186/s12883-022-02745-8

**Published:** 2022-06-13

**Authors:** Daisuke Ito, Michiyuki Kawakami, Ryota Ishii, Masahiro Tsujikawa, Kaoru Honaga, Kunitsugu Kondo, Tetsuya Tsuji

**Affiliations:** 1 Department of Rehabilitation Medicine, Keio University School of Medicine, 35 Shinanomachi, Shinjuku-ku, Tokyo, 160-8582 Japan; 2 Department of Rehabilitation Medicine, Tokyo Bay Rehabilitation Hospital, 4-1-1, Yatsu, Narashino City, Chiba, 275-0026 Japan; 3 Department of Biostatistics, Faculty of Medicine, University of Tsukuba, 1-1-1, Tennodai, Tsukuba, Ibaraki 305-8577 Japan; 4 Department of Rehabilitation Medicine, Juntendo University Graduate School of Medicine, 2-1-1, Hongo, Bunkyo-ku, Tokyo, 113-8421 Japan

**Keywords:** Cognitive function, Cognitive impairment, Discharge destination, Retrospective cohort study, Stroke

## Abstract

**Aim:**

To investigate the cognitive function and its relation to the home discharge of patients following subacute stroke.

**Methods:**

This retrospective cohort study included 1,229 convalescent patients experiencing their first subacute stroke. We determined discharge destination and demographic and clinical information. We recorded the following measurement scores: Mini-Mental State Examination (MMSE) score, Stroke Impairment Assessment Set score, grip strength, and Functional Independence Measure (FIM). We performed a multivariable logistic regression analysis with the forced-entry method to identify factors related to home discharge.

**Results:**

Of the 1,229 participants (mean age: 68.7 ± 13.5 years), 501 (40.8%), 735 (59.8%), and 1,011 (82.3%) were female, had cerebral infarction, and were home discharged, respectively. Multivariable logistic regression analysis revealed that age (odds ratio [OR], 0.93; 95% confidence interval [CI], 0.91 – 0.96; *P* < 0.001), duration from stroke onset to admission (OR, 0.98; 95% CI, 0.96 – 0.99; *P* = 0.003), living situation (OR, 4.40; 95% CI, 2.69 – 7.20; *P* < 0.001), MMSE score at admission (OR, 1.05; 95% CI, 1.00 – 1.09; *P* = 0.035), FIM motor score at admission (OR, 1.04; 95% CI, 1.01 – 1.06; *P* = 0.001), and FIM cognitive score at admission (OR, 1.08; 95% CI, 1.04 – 1.13; *P* < 0.001) were significantly associated with home discharge.

**Conclusions:**

MMSE at admission is significantly associated with home discharge in patients with subacute stroke.

**Supplementary Information:**

The online version contains supplementary material available at 10.1186/s12883-022-02745-8.

## Background

Stroke is a leading cause of disability [1, 2]. Patients with mild stroke in the acute phase are usually discharged within a short period after stroke onset [3]. However, patients who need assistance in activities of daily living (ADL) after acute treatment require intensive rehabilitation. In Japan, subacute stroke patients still assisted in ADL after treatment in acute hospitals have been transferred to the convalescent rehabilitation wards and have undergone intensive rehabilitation since 2000 [4]. From 2000 to 2006, patients were admitted within three months of stroke onset as subacute stroke patients; from 2006 to 2020, within two months [4]; and after 2020, no longer depends on the duration from stroke onset. In convalescent rehabilitation wards, the maximum length of stay covered by the insurance is 150 days for stroke, 180 days for stroke with severe disability and cognitive disorders, and the maximum rehabilitation time for stroke patients is 3 h per day, including weekends (21 h per week) [4]. Subacute stroke patients who are admitted to convalescent rehabilitation wards undergo rehabilitation to improve their ADL or return to their homes [4]. Discharge planning for patients is a vital topic in subacute stroke rehabilitation. Appropriate discharge destination planning for inpatients following a stroke can enhance reasonable use of healthcare resources, improve clinical outcomes, and decrease the financial burden of patients [5]. Thus, in the rehabilitation pipeline for subacute stroke patients, accurate prediction of the possibility of home discharge from the early stage of hospitalization is important.

Previous studies have reported factors related to home discharge in patients with subacute stroke with an onset of about 30 days [6–18]. In particular, many studies have consistently suggested that functional disability is related to home discharge. In a meta-analysis, for every 1-point increase in the Functional Independence Measure (FIM), a stroke patient was 1.08-times more likely to be discharged home than to institutionalized care [6]. Moreover, in a systematic review, marital status and social support were associated with the discharge destination [7]. Therefore, functional disability and social factors are essential factors for predicting home discharge. Additionally, demographic characteristics such as age [8], sex [9], and duration of hospitalization [10] were associated with home discharge. However, few studies have predicted home discharge based on the severity of post-stroke impairments, such as physical function [11, 17] and cognitive impairment [8]. Therefore, it is necessary to investigate these various factors, including common post-stroke impairments.

Cognitive impairment is a common symptom in patients with stroke. The prevalence of cognitive impairment within one-year post-stroke was 38%, according to a systematic review [19]. Moreover, post-stroke cognitive impairment has been reported to be associated with dependency [20] and increased costs for utilization of care [21]. However, to date, no studies have investigated the relationship between home discharge and general cognitive impairment in subacute stroke patients by multivariable analysis.

Therefore, this study aimed to explore the factors associated with home discharge in subacute stroke patients, adding cognitive function to other factors reported in previous studies such as FIM, social factors, demographic characteristics, and physical function.

## Methods

### Study design and participants

**Fig. 1:**
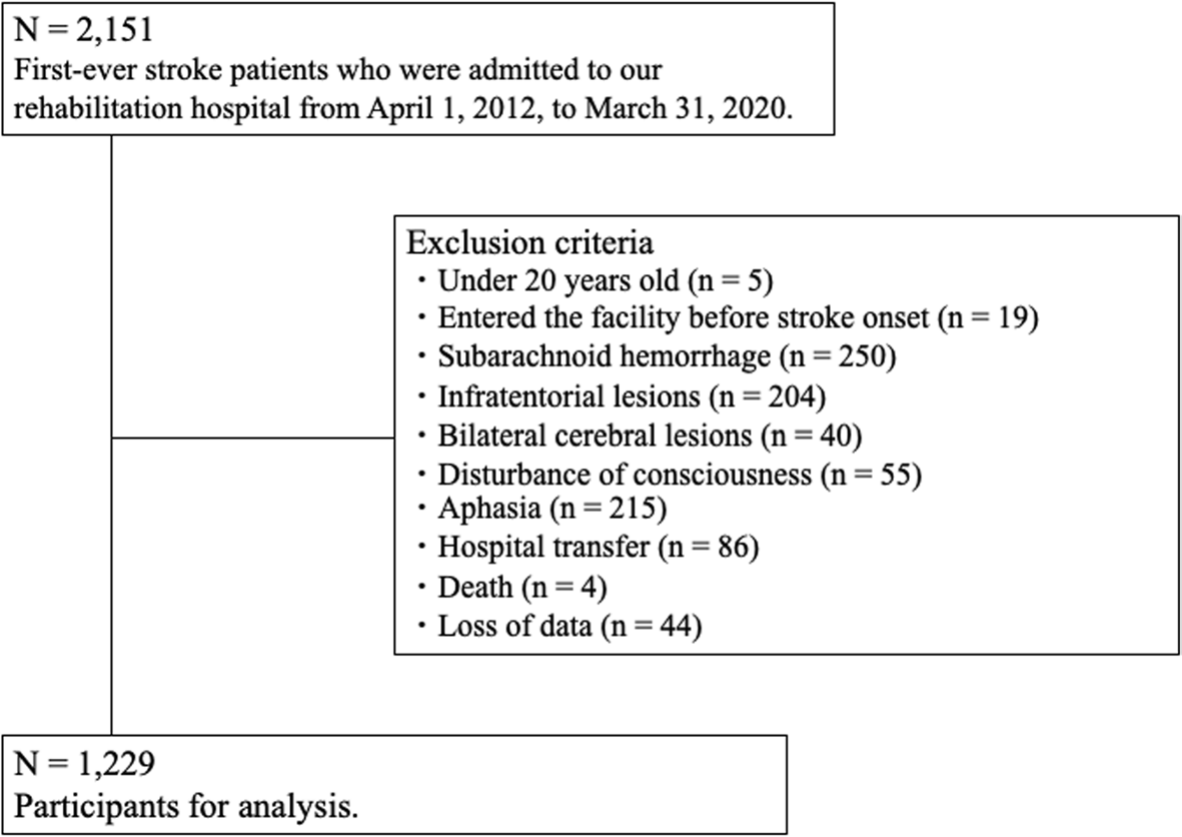
Flowchart of the patient selection process

This retrospective cohort study was reported in adherence to the STROBE statement. This study collected 2,151 consecutive patients with subacute stroke admitted to Tokyo Bay Rehabilitation Hospital between April 1, 2015 and March 31, 2020. The inclusion criterion was a first occurrence of subacute stroke. The exclusion criteria were age < 20 years (*n* = 5), entered the facility before stroke onset (*n* = 19), subarachnoid hemorrhage (*n* = 250), infratentorial lesions (*n* = 204), bilateral cerebral lesions (*n* = 40), disturbance of consciousness (*n* = 55), aphasia (*n* = 215), hospital transfer (*n* = 86), death (*n* = 4), and loss of data (*n* = 44). After applying the selection criteria, 1,229 patients were finally included in this study (Fig. 1). This study was conducted in accordance with the Declaration of Helsinki [22] and was reviewed and approved by the Ethics Committee of Tokyo Bay Rehabilitation Hospital (approval number #246). The opt-out method was applied to obtain informed consent in this study.

### Setting

Tokyo Bay Rehabilitation Hospital is a subacute rehabilitation ward with 160 beds. All patients in this study completed a rehabilitation program for 120 – 180 min a day during the hospitalization, including ≥ 60 min of physical therapy, ≥ 60 min of occupational therapy, and/or ≥ 40 min of speech-language-hearing therapy.

### Data collection

The following demographic characteristics and measures were collected from the patients’ medical records by the first author: age, sex, body mass index (BMI), stroke type (cerebral infarction or cerebral hemorrhage), brain side affected, duration from stroke onset to admission, hospital duration, living situation (alone or not), and discharge destination (home or facility). Hospital duration and discharge destination were collected at discharge, while the other data were collected at admission.

### Mini-mental state examination

Mini-Mental State Examination (MMSE) is a questionnaire for evaluating cognitive function [23]. It consists of 11 items as follows (maximum score of each item): orientation to time (5), orientation to place (5), registration of three words (3), attention and calculation (serial sevens or spelling) (5), recall (3), naming (2), repetition (1), comprehension of verbal (3), comprehension of written (1), writing (1), and construction (1). The maximum score is 30 points, with a higher score representing greater cognitive function; the cut-off value is 23 points [23]. Occupational therapists administered the MMSE and determined the score at admission.

### Stroke impairment assessment set

Motor function was assessed using the stroke impairment assessment set-motor function (SIAS-m) [24, 25], which consists of two tests for the upper extremity (knee-mouth and finger function tests) and three tests for the lower extremity (hip flexion, knee extension, and foot pat tests). Each test was rated on a 6-grade ordinal scale rating from 0 (no movement at all) to 5 points (normal). The total scores of the upper and lower extremities were 0 – 10 and 0 – 15 points, respectively [26]. Physical and occupational therapists administered the SIAS-m and determined the score at admission.

### Grip strength

Upper-body muscle strength was measured using grip strength, which has established reliability in patients with stroke [27]. Grip strength was measured for each participant’s non-paralyzed upper limb using a handgrip dynamometer (TKK 5401; Takei Scientific Instruments, Tokyo, Japan). Representative grip strength was calculated as the average of two trials [28]. Each measurement was assessed by trained physical or occupational therapists.

### Functional independence measure

FIM version 3.0 is an observational evaluation tool for functional disability [29]. The FIM consists of 13 motor subscales (FIM motor) and five cognitive subscales (FIM cognitive). The FIM motor consists of the following four categories: self-care (eating, grooming, bathing, dressing-upper body, dressing-lower body, and toileting), sphincter control (bladder management and bowel management), transfers (bed/chair/wheelchair, toilet, and tub/shower), and locomotion (walk/wheelchair and stairs). The FIM cognitive consists of two categories: communication (comprehension and expression) and social cognition (social interaction, problem-solving, and memory). Each item has a 7-grade scale ranging from 1 (total assistance or not testable) to 7 points (complete independence). The total score is 18 – 126 points, 13 – 91 points, and 5 – 35 points for the total FIM, FIM motor, and FIM cognitive, respectively, with a higher score representing greater functional independence. Nurses evaluated FIM scores at admission and discharge.

### Statistical analyses

The normality of continuous variables was assessed using the normal Q-Q plot. Patient characteristics were summarized for the home and facility discharge groups and compared between groups using the chi-squared test, unpaired t-test, or Mann–Whitney U test, as appropriate. Finally, a multivariable logistic regression analysis with the forced-entry method was performed to assess the factors affecting home discharge after controlling simultaneously for potential confounders. The dependent variable was the discharge destination (home or facility), and the independent variables were all factors at admission. The multicollinearity of the independent variables was assessed using the variance inflation factor. Multicollinearity is present when the variance inflation factor is higher than 5 to 10 [30]. Furthermore, we tested the validity of our model using a Hosmer–Lemeshow test and the percentage of correct classifications. All statistical analyses were performed using IBM SPSS Statistics (version 27.0; IBM, Tokyo, Japan). Statistical significance was set at P ≤ 0.05.

## Results

The characteristics of the study participants are listed in Table 1. The mean age ± standard deviation of the 1,229 patients with stroke was 68.7 ± 13.5 years. There were 1,011 participants (82.3%) in the home discharge group and 218 participants (17.7%) in the facility discharge group. Male sex, cerebral infarction, right brain side being affected, and not living alone were factors more likely to be associated with the home discharge group; these patients also had a younger age, shorter duration from stroke onset to admission, shorter hospital duration, and higher BMI, MMSE score, SIAS-m score, grip strength, and FIM score than those in the facility discharge group (*P* < 0.050).

**Table 1:** Characteristics of the study participants

	Overall	Home	Facility	*P* value
	*n* = 1229	*n* = 1011	*n* = 218	
Age, y^a^	68.7 (13.5)	66.5 (13.3)	78.7 (8.9)	** < 0.001**
Sex (men)^b^	728 (59.2)	645 (63.8)	83 (38.1)	** < 0.001**
BMI, kg/m^2 a^	21.8 (3.2)	22.2 (3.2)	20.4 (3.1)	** < 0.001**
Stroke type (cerebral infarction)^b^	735 (59.8)	589 (58.3)	146 (67.0)	**0.017**
Brain side affected (right)^b^	667 (54.3)	530 (52.4)	137 (62.8)	**0.005**
Duration from stroke onset to admission^a^	32.2 (12.7)	31.1 (12.3)	37.2 (13.5)	** < 0.001**
Hospital duration^a^	88.5 (45.3)	82.4 (44.9)	116.9 (35.6)	** < 0.001**
Living circumstance (alone)^b^	225 (18.3)	157 (15.5)	68 (31.2)	** < 0.001**
MMSE score at admission^a^	23.5 (6.2)	24.8 (5.3)	17.4 (6.6)	** < 0.001**
Grip strength at admission, kgf^a^	24.2 (10.5)	26.1 (10.0)	15.2 (8.4)	** < 0.001**
SIAS-m U/E score at admission (0–10) ^c^	7 (3–8)	8 (3–9)	3 (0–8)	** < 0.001**
SIAS-m L/E score at admission (0–15)^c^	12 (6–13)	12 (8–14)	6 (1–12)	** < 0.001**
FIM motor score at admission^c^	51 (32–68)	56 (40–71)	24 (17–37)	** < 0.001**
FIM cognitive score at admission^c^	26 (20–31)	28 (23–32)	17 (13–22)	** < 0.001**
FIM total score at admission^c^	78 (54–96)	84 (65–101)	42 (32–57)	** < 0.001**
FIM motor score at discharge^c^	82 (67–88)	85 (76–88)	48 (34–65)	** < 0.001**
FIM cognitive score at discharge^c^	31 (25–35)	32 (28–35)	22 (17–27)	** < 0.001**
FIM total score at discharge^c^	112 (93–121)	116 (105–122)	70 (52–91)	** < 0.001**

^a^Mean (standard deviation), ^b^number (%), ^c^ median (interquartile range)

*Abbreviations*: *BMI* body mass index, *FIM* Functional Independence Measure, *MMSE* Mini-Mental State Examination, *SIAS-m* Stroke Impairment Assessment Set-motor function, *U/E* upper extremity, *L/E* lower extremity

Multivariable logistic regression analysis was performed to identify variables associated with home discharge (Table 2). The factors at admission significantly associated with home discharge were age (odds ratio [OR], 0.93; 95% confidence interval [CI], 0.91 – 0.96; *P* < 0.001), duration from stroke onset (OR, 0.98; 95% CI, 0.96 – 0.99; *P* = 0.003), living situation (OR, 4.40; 95% CI, 2.69 – 7.20; *P* < 0.001), MMSE score (OR, 1.05; 95% CI, 1.00 – 1.09; *P* = 0.035), FIM motor score (OR, 1.04; 95% CI, 1.01 – 1.06; *P* = 0.001), and FIM cognitive score (OR, 1.08; 95% CI, 1.04 – 1.13; *P* < 0.001). There were no factors with variance inflation rate ≥ 5. The Hosmer–Lemeshow test shows *P* = 0.944 and the percentage of correct classification is 88.3%, which indicates a good fit for the regression model.

**Table 2:** Multivariable logistic regression analysis of the home discharge

Variable at admission	OR	95% CI		*P* value	VIF
		Lower	Upper		
Age	0.93	0.91	0.96	** < 0.001**	2.06
Sex					
Women	0.98	0.57	1.69	0.947	1.94
Men (reference)	Reference				
BMI	1.03	0.96	1.10	0.431	1.23
Stroke type					
Cerebral hemorrhage	1.17	0.73	1.85	0.519	1.29
Cerebral infarction (reference)	Reference				
Brain side affected					
Left	1.38	0.90	2.12	0.144	1.09
Right (reference)	Reference				
Duration from stroke onset to admission	0.98	0.96	0.99	**0.003**	1.08
Living circumstance					
Not alone	4.40	2.69	7.20	** < 0.001**	1.03
Alone (reference)	Reference				
MMSE score	1.05	1.00	1.09	**0.035**	2.42
Grip strength	1.03	0.99	1.06	0.166	3.13
SIAS-m U/E score	1.03	0.93	1.15	0.560	3.63
SIAS-m L/E score	1.07	0.99	1.16	0.105	4.17
FIM motor score	1.04	1.01	1.06	**0.001**	4.16
FIM cognitive score	1.08	1.04	1.13	** < 0.001**	2.81

Model χ^2^ test *P* < 0.001, Hosmer and Lemeshow test *P* = 0.944, percentage of correct classifications: 88.3%

Dependent variable: discharge destination (reference, facility)

*Abbreviations*: *BMI* body mass index, *CI* confidence interval, *FIM* Functional Independence Measure, *L/E* lower extremity, *MMSE* Mini-Mental State Examination, *OR* odds ratio, *SIAS-m* Stroke Impairment Assessment Set-motor function, *U/E* upper extremity, *VIF* variance inflation factor

## Discussion

We investigated factors associated with home discharge in patients with subacute stroke. Multivariable logistic regression analysis revealed that age, duration from stroke onset to admission, living situation, MMSE score at admission, FIM motor score at admission, and FIM cognitive score at admission were significantly associated with home discharge.

The MMSE score at admission was significantly associated with home discharge. While a previous study also reported that the MMSE score is associated with home discharge [8], the examination was limited to univariate analysis. To date, this is the first study to investigate the relationship between home discharge and MMSE score for stroke patients in a multivariable analysis. We found a significant association between home discharge and MMSE score, even after adjusting for factors associated with home discharge. The MMSE may be a predictor of home discharge in subacute stroke patients. Therefore, assessing the MMSE at admission in the subacute phase can lead to appropriate discharge support following intensive rehabilitation.

Furthermore, it was shown that besides the MMSE, the FIM cognitive score was also associated with home discharge. Many previous studies have reported on the association between FIM cognitive score and discharge [9, 11, 12, 17]. Although both are cognitive assessments, the MMSE evaluates cognitive impairment such as that affecting memory, attention, and executive function, and the FIM cognitive scale evaluates cognitive disability in ADL. Specifically, the severity of cognitive impairment and amount of assistance related to cognitive disability affect home discharge independently. For example, a previous study of Alzheimer's disease reported that the severity of cognitive impairment did not correlate with the severity of burden; instead, anosognosia and behavioral abnormalities are associated with care burden [31]. Similarly, in stroke patients, it is essential to evaluate cognitive function from the functional and ADL aspects to predict home discharge accurately.

Multivariable logistic regression analysis revealed that age, duration from stroke onset to admission, living situation, and FIM motor score at admission were also associated with home discharge in subacute stroke patients. Previous studies have reported the association between discharge and age [8], duration from stroke onset to admission [14], social factors [7–9, 11, 15, 17], and FIM score [6, 8, 10, 16]; these findings are consistent with our findings. Therefore, it is essential to prepare for home discharge by assessing cognitive function and considering age, social factors, and ADL ability at admission in subacute stroke patients.

The strength of this study is the use of large-scale data to comprehensively identify factors associated with home discharge of subacute stroke patients, including demographic characteristics, functional impairment, and disability. Investigation of factors associated with home discharge requires large-scale data studies to consider confounding factors. Thus, the results of this study, using large-scale data and including functional outcomes such as SIAS-m score, grip strength, and MMSE score, are important findings regarding the rehabilitation of subacute stroke patients.

However, this study had some limitations. First, we used the MMSE scores to determine cognitive impairment; thus, we excluded patients with disturbance of consciousness and aphasia. Cognitive function may be associated with home discharge, even in patients with aphasia. Thus, future studies using nonverbal cognitive assessments are needed. Similarly, we used the SIAS-m scores to determine motor function; thus, we excluded patients with bilateral cerebral lesions. The inclusion of patients with bilateral motor paralysis may reveal different associated factors compared to this study. Second, data related to the location of the brain lesion, such as stroke subtypes, region, volume, or dominance, were not collected. Several previous studies have reported that stroke subtypes are associated with home discharge; therefore, including them may improve the accuracy of the analysis. Third, the severity of stoke was not examined. In the acute phase, stroke severity, such as the National Institutes of Health Stroke Scale, may be useful for home discharge. However, our study includes MMSE, SIAS, and FIM, making for similar consideration. Finally, the study was conducted in a single facility, which limits the generalizability of our results. Despite these limitations, the findings of this study are valuable as they suggest that the MMSE is a useful predictor of home discharge in subacute stroke patients. The MMSE is widely and commonly used for subacute stroke patients; hence, the MMSE can be a useful tool for such patients. In the future, it will be necessary to investigate whether interventions for cognitive dysfunction and higher brain dysfunction can improve return-to-home rates.

## Conclusion

The current study revealed that age, duration from stroke onset to admission, living situation, MMSE score at admission, FIM motor score at admission, and FIM cognitive score at admission were significantly associated with home discharge in subacute stroke patients who were undergoing rehabilitation in convalescent wards. Among them, the significant association between MMSE score and home discharge is a novel finding. Therefore, screening for cognitive function on admission in patients with subacute stroke is important.

## Supplementary Information

**Additional file 1. **

**Additional file 2. **

**Additional file 3. **

## Data Availability

The datasets used and/or analyzed during the current study are available from the corresponding author on reasonable request.

## Abbreviations

BMI: Body mass index
CI: Confidence interval
FIM: Functional Independence Measure
MMSE: Mini-Mental State Examination
OR: Odds ratio
SIAS-m: Stroke Impairment Assessment Set-motor function
